# Editorial: Exploring regenerative pathways in muscle repair

**DOI:** 10.3389/fphys.2025.1665619

**Published:** 2025-07-29

**Authors:** Jeffrey Scott Otis, Barbara St. Pierre Schneider, Paula Tavares

**Affiliations:** ^1^ Georgia State University, Atlanta, GA, United States; ^2^ Department of Graduate Nursing, The University of Texas at Arlington, Arlington, TX, United States; ^3^ Universidade de Coimbra, Faculty of Sport Sciences and Physical Education, Coimbra, Portugal

**Keywords:** muscle physiology/performance, muscle regeneration, skeletal muscle health, muscle repair mechanisms, muscle growth and function

## 1 Introduction

Muscle regeneration is a tightly controlled biological process that involves cellular, metabolic, and mechanical pathways working together to restore tissue health and function. Disruption of these pathways—whether through illness, disuse, trauma, or chemical damage—can lead to long-term dysfunction in strength, metabolism, and impact overall quality of life. The Research Topic “Exploring Regenerative Pathways in Muscle Repair” features four innovative works that collectively enhance our understanding of muscle regeneration, ranging from the molecular aspects of disease to biomechanical and physiological methods that promote recovery.

## 2 Blood flow restriction exercise as a regenerative catalyst in intensive care unit-acquired weakness

The opening study addresses one of the most difficult clinical challenges in muscle rehabilitation: intensive care unit-acquired weakness (ICUAW). Characterized by long-term muscle loss and decreased satellite cell activity, ICUAW remains a persistent complication even years after hospitalization. Mathur et al. tested a new low-intensity, short-duration blood flow restriction exercise (BFRE) protocol designed to promote muscle regeneration.

Among healthy individuals, the BFRE protocol increased satellite cell content and altered gene expression in ways that suggest muscle remodeling, as evidenced by the upregulation of MuRF1 and downregulation of myostatin. Notably, ICUAW survivors showed mixed responses. One survivor with preserved muscle mass responded positively to BFRE, while those with significant atrophy exhibited no change in satellite cell content.

These findings highlight a crucial reliance on residual regenerative capacity and satellite cell function. This study offers promising translational insights, presenting BFRE as a practical and effective method for initiating regeneration, particularly for those who cannot perform resistance exercise.

## 2 Biomechanical recovery via contrast therapy: A comparative assessment

Shifting focus from a clinical population to high-performance athletes, the second study examines the biomechanical effects of contrast water immersion therapy (CWT) versus Game Ready contrast therapy (GRT) on muscle tone, stiffness, elasticity, pain threshold, and strength in mixed martial arts (MMA) athletes. These athletes engage in high-intensity exercise, which predisposes to sustaining an acute muscle injury (Zebrowska et al., 2019). In a randomized controlled trial, Trybulski et al. showed that both GRT and CWT resulted in nearly identical improvements in all measured variables, except for muscle tone, where GRT had a more potent effect.

These findings confirm both therapies as effective recovery approaches and suggest a preference for GRT in situations where muscle tone regulation is essential. Muscle tone, a key factor in performance, injury prevention, and neuromuscular coordination, may respond more strongly to GRT’s mechanical compression features.

The physiological rationale appears to lie in the temperature and pressure fluctuations inherent to contrast therapy, which promote perfusion, reduce inflammation, and facilitate the clearance of metabolites. This study offers practical insights for the field of sports medicine and rehabilitation, demonstrating that non-invasive, relatively passive modalities can significantly influence key biomechanical markers of muscle function and readiness.

## 3 Hyperaemia and perfusion specificity in contrast compression therapy

Building on contrast therapy mechanisms, Trybulski et al., investigate vascular perfusion responses, utilizing the same GRT modality to assess hyperemic reactions in MMA athletes. The research shows a significant increase in blood flow in the treated limb, with no notable change in the opposite limb, indicating that GRT-induced hyperemia is localized and not influenced systemically or reflexively.

Trybulski et al. establish that peripheral blood flow enhancement is a targeted physiological response, which may be critical for optimizing recovery from localized muscle fatigue or trauma. Secondly, they highlight the importance of precise application strategies in both clinical and athletic settings. Blanket assumptions about systemic circulatory benefits from localized treatment may require reconsideration.

Furthermore, the extended period for blood flow normalization after treatment suggests that GRT may cause a prolonged vasodilatory state, which could be utilized to enhance nutrient delivery and metabolic clearance following exercise. Overall, this study helps refine the physiological understanding of contrast therapy by aligning vascular response data with muscle performance outcomes.

## 4 Lipid dysregulation as a potential target for ameliorating chronic alcohol-related myopathy

In the final article, Ganjayi et al. present a mechanistic hypothesis in the field of chronic alcohol-related myopathy (CAM) and reinterprets the disorder not just as a result of widespread toxicity but as one caused by lipid dysregulation. This literature-based analysis combines human and animal studies to suggest that changes in lipid composition—specifically, intramuscular lipid buildup and signaling—may be a key factor in ethanol-induced muscle atrophy.

This emerging perspective unlocks new investigative and therapeutic possibilities. Lipids are not only structural or energy sources, but also serve as signaling molecules that influence muscle growth, inflammation, and apoptosis. Disrupted lipid profiles may hinder anabolic signaling pathways (e.g., mTOR) while activating catabolic systems (e.g., the ubiquitin-proteasome or autophagy-lysosome pathways), leading to the gradual loss of muscle tissue.

If lipid modulation proves to be causal, then interventions targeting lipid metabolism—such as PPAR agonists, omega-3 fatty acid supplementation, or mitochondrial-targeted therapies—could become effective CAM treatments. This review encourages the field to look beyond muscle fiber architecture and focus on bioenergetic and metabolic factors contributing to muscle regeneration failure.

## 5 Conclusion

Together, compelling themes emerge from these four studies:

1. Muscle regeneration depends on biological readiness. Whether related to an ICU experience or chronic alcohol use, underlying cellular or metabolic problems may restrict the success of well-designed treatments.
2. Muscle-enhancing therapy must be tailored to the individual’s physiological situation. BFRE may be an option for persons exhibiting frailty, while contrast therapies for highly trained individuals recovering from performance.
3. Perfusion and muscle recovery are closely linked. Increased blood flow, whether through exercise or thermal contrast, consistently correlates with muscle repair and function.
4. The metabolic environment is important. Lipid dysregulation in CAM underscores that muscle regeneration depends not only on muscle cells but also on the broader metabolic context in which they operate.

This Research Topic highlights the need for multidimensional approaches—combining molecular biology, biomechanics, and physiology—to better understand and improve muscle regeneration. Future research should continue to connect mechanistic insights with practical applications, advancing therapies that are both scientifically sound and functionally effective.
